# Left ventricular post-infraction pseudoaneurysm mimicking mitral valve endocarditis

**DOI:** 10.1186/1749-8090-8-211

**Published:** 2013-11-15

**Authors:** Panagiotis Dedeilias, Ioannis Koukis, Antonios Roussakis, Pantelis Tsipas, Effie Rouska

**Affiliations:** 1 Cardiac surgery Department, Evangelismos Hospital, Athens, Greece; 2 Cardiology Department, Evangelismos Hospital, Athens, Greece

**Keywords:** Pseudoaneurysm, Aorta, Mitral valve, Dor procedure

## Abstract

In this report we present a patient who was initially diagnosed as suffering from mitral valve endocarditis. The proper use of diagnostic modalities revealed a pseudo aneurysm of the left ventricle which was mimicking mitral valve vegetations. This allowed better planning of the subsequent operation. The optimal preoperative diagnostic studies are discussed along with the proper surgical treatment.

## Background

Left ventricular pseudoaneurysm is a rare condition associated with a high risk of rapid enlargement and rupture. They are rarely suspected at clinical presentation and their symptoms are non specific. In this case report, a large pseudo aneurysm of the left ventricle (LV) was initially mistaken for mitral valve vegetations. We report the optimal preoperative studies as well as the operative management and review of the literature.

## Case presentation

A 74 year-old Caucasian male was admitted elsewhere due to persistent fever. During his stay he sustained an acute coronary syndrome. A subsequent coronary angiogram revealed a 90% left anterior descending (LAD) stenosis and a 100% right coronary artery (RCA) occlusion. Both were addressed with drug eluted stents and the patient was discharged two days later.

**Figure 1:**
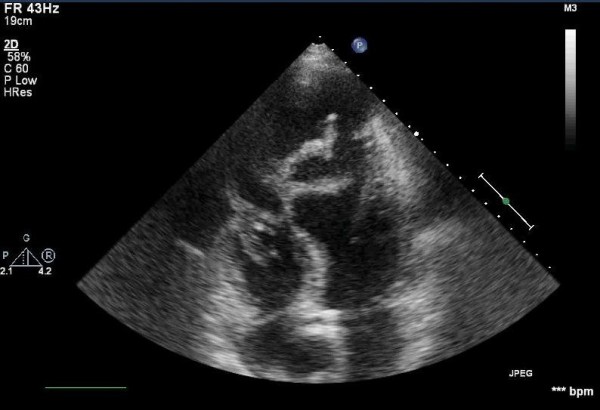
Transthoracic echo showing the peculiar looking mitral valve.

Since the fever persisted, he was admitted 4 days later to the General ward of our Hospital. Blood cultures were positive to enterococcus faecalis and the appropriate antibiotic treatment was commenced. Whilst still on diagnostic workup the patient had a transthoracic echocardiogram (TTE) which revealed marginal normal dimensions of the left ventricle with a reported ejection fraction ranged between 30–35%. There was no evidence of left ventricular thrombus but there was significant mitral valve regurgitation with vegetations onto the posterior mitral leaflet (Figure 1).

Nine days later despite the appropriate medical treatment, the patient’s condition deteriorated and he became unstable with need for inotropic support.

A second transthoracic echocardiogram revealed a surprising finding. The aforementioned significant mitral valve regurgitation with vegetations was in fact a large pseudo aneurysm of the left ventricle (6.5 × 6.0 cm) with remarkable movement of the involved ruptured apical wall mimicking the movements of the mitral leaflets, and concomitant moderate mitral valve regurgitation. A new transoesophageal echo (TOE) as well as a CT scan confirmed the diagnosis of a left ventricular pseudo aneurysm rather than mitral valve endocarditis.

**Figure 2:**
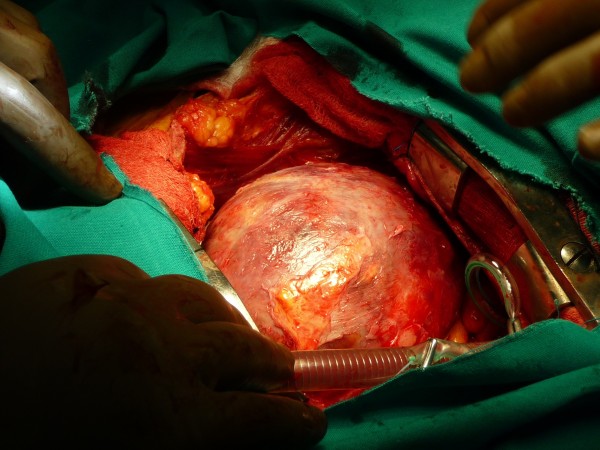
Intraoperative view of the pseudo aneurysm.

**Figure 3:**
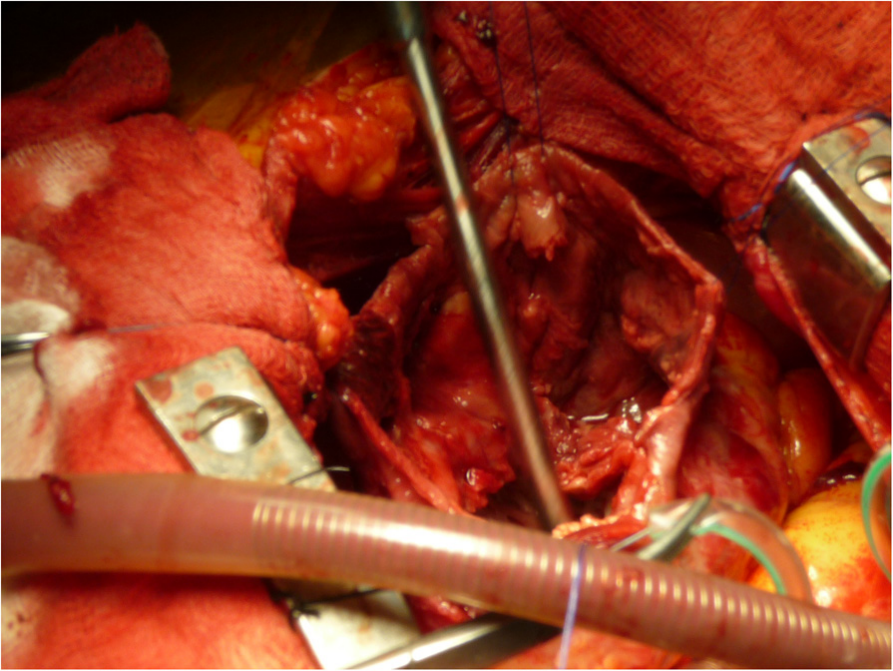
The pseudo aneurysm opened.

**Figure 4:**
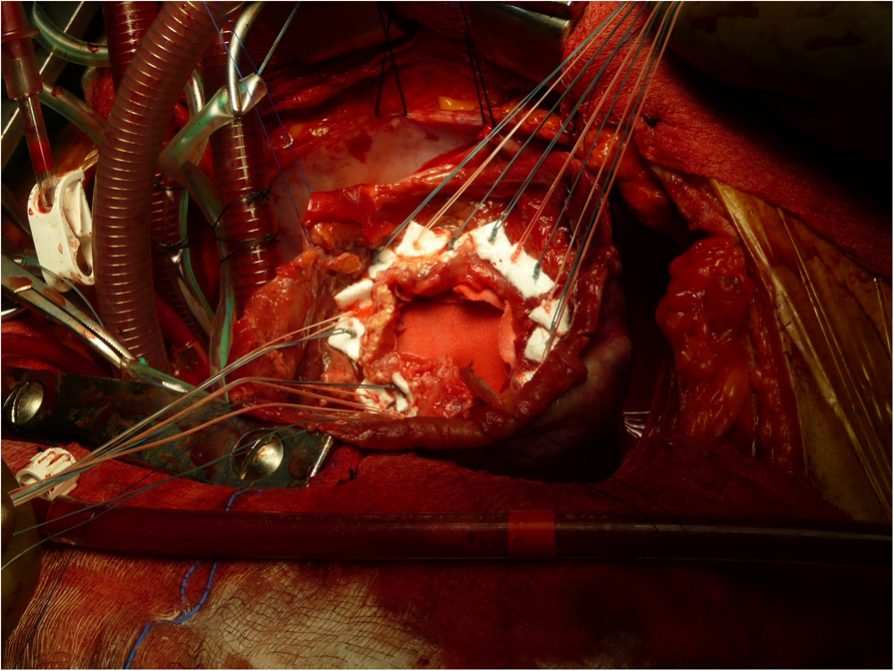
Dor procedure almost completed.

Immediately after, a left ventriculoplasty (Dor procedure) was offered. After median sternotomy the pericardium was opened revealing abundant blood-tinged pericardial fluid and fresh, thick adhesions. After careful dissection of the pseudoaneurysm (Figure 2), cardiopulmonary bypass was initiated through standard aortic and right atrial cannulation. After cardioplegic arrest and left ventricular venting through the right superior pulmonary vein, the pseudoaneurysm of the apex was opened. The aneurysmatic chamber’s diameter was estimated to be 6 cm (Figure 3). Necrotic tissue was excised and the defect was closed with a synthetic Dacron patch sutured with interrupted pledgetted mattress sutures (Ti cron 2–0™) (Figure 4). The aortic cross clamp time removed after 40 min and the heart took over the circulation fairly easily with modest inotropic support. Total bypass time was 72 min run.

His post-operative course was unremarkable except from transient renal insufficiency treated with sessions of haemofiltration and minor pulmonary atelectasis treated with the appropriate chest physiotherapy. Left ventricular diameter and movement was back to normal limits. Ejection fraction was back up to 55%. His temperature gradually declined and three consequent blood cultures were negative. He was discharged on an ambulatory setting on the 24th postoperative day and remains well in one year follow-up.

## Discussion

Left ventricular pseudo aneurysm (LVPA) (or false aneurysm) is a rare condition and forms when cardiac rapture is contained by pericardial adhesions or fiber tissue. The most common cause of LV pseudoaneurysm is myocardial infarction (MI) (over 50%) followed by previous heart surgery (about 1/3 of the cases), trauma (over 5%) and infection (about 5%). Post-infraction pseudo aneurysms (PIPA) are characterized as acute when they are diagnosed within the first two weeks from the infraction and as chronic when they are discovered later than a 14-day period from the event. The clinical presentation may vary depending upon congestive heart failure, mitral regurgitation, ventricular tachy-arrhythmia, systemic thrombo-embolism and cardiac rupture [1,2]. In general, patients do not have specific symptoms pertaining to pseudo aneurysm [1], hence the diagnosis may be delayed. Wall stress, which is related to LV pressure and radius, and loss of myocardial integrity, because of the infraction, are the most probable reasons of cardiac rapture. Pathologically, LVPA have a narrow neck [3,4] that connects the heart chamber with a large aneurismal sac, which contains blood and thrombus and is lined by fibrous pericardial tissue with no myocardial elements. On the contrary, true post-infraction aneurysms have a larger point of entry caused by scar formation which results in thinning of the myocardium [3]. Angiography, transthoracic or transoesophageal echocardiography, CT-scan and MRI are the imaging modalities which are used in the diagnostic process of LVPA, with the first two being the most reliable and frequently used [3,5,6].

When a LVPA is discovered, surgical repair is performed in most cases. Surgery is the recommended treatment for PIPAs diagnosed within the first sixty to ninety days after the myocardial infarction, because there is high possibility of rupture. On the other hand, when the discovery of a LVPA is made months or even years after the infarction, surgery is not the treatment of choice [3,4]. Left ventricular pseudo aneurysms can be repaired with various techniques depending on the time interval between the infarction and LVPA’s diagnosis. Chronic pseudo aneurysms can be repaired by direct suturing of the neck with horizontal mattress sutures buttressed with Teflon felt strips and a patch secured over the repair. In acute cases, epicardial repair can be performed by placement of a patch of pericardium, Dacron, or polytetrafluoroethylene (PTFE) over the ventricular defect; the patch is sutured to healthy myocardium along the periphery of the infarct. In the case discussed here the LVPA occurred as a result of rupture of the LV and the wall was composed of only the epicardium along with clots and fibrin. Thus the treatment of choice was the exclusion and synthetic patch replacement as in Dor procedure. Direct over sewing with buttress sutures was not an option due to tissue friability. False aneurismal wall can either be removed or sewn over the patch [4].

Giant LV pseudo aneurysm has been reported to cause mitral regurgitation and compression of adjacent vascular structures [7-9]. However, in the present case the aneurysm was actually mistaken for an insufficient mitral valve with vegetations. This impression is by all means uncommon and to our knowledge unique in the relevant literature. The febrile and septic status of the patient might have been a potential misleading factor; the position of the false lumen another. The second TTE along with the TOE revealed the actual LVPA and excluded the initial impression of the infected mitral valve (MV). Thus, in such cases of suspected MV endocarditis the additional confirmation with the TOE should always be encouraged. In the present case, relying on the first TTE for planning of the operation would have had catastrophic consequences for the patient. Once the diagnosis of LVPA is confirmed, further delineation can be obtained via CT scanning.

## Conclusion

In conclusion, LVPA are rare conditions which can sometimes be mistaken for other cardiac diseases. Careful patient workup with aid of the TOE and confirmation with CT scanning can reveal the LVPA and help in planning the necessary surgical correction. Surgery is the definitive treatment of giant pseudo aneurysms, without which the prognosis is very poor.

## Consent

Written informed consent was obtained from the patient for publication of this Case report and any accompanying images. A copy of the written consent is available for review by the Editor-in-Chief of this journal.

## Abbreviations

LV: Left ventricle; LAD: Left anterior descending; RCA: Right coronary artery; LVPA: Left ventricular pseudo aneurysm; PIPA: Post-infraction pseudo aneurysms; PTFE: Polytetrafluoroethylene; TTE: Transthoracic echo; TOE: Transoesophageal echo; MV: Mitral valve.

## Competing interests

The author(s) declare that they have no competing interests.

## Authors’ contributions

PD, IK, PT, AR, operated on patient and prepared the manuscript. ER, did the preop workup. All authors read and approved the final manuscript.
